# Design of an eccentric recumbent ergometer to elicit delayed onset muscle soreness

**Published:** 2021-04-15

**Authors:** Sara A. Harper, Frederick J. Peters, Brandon S. Pollock, Keith Burns, John McDaniel, Angela L. Ridgel

**Affiliations:** 1 Department of Kinesiology and Health Science, Utah State University, Logan, UT, USA; 2 Sorenson Legacy Foundation Center for Clinical Excellence, Utah State University, Logan, UT, USA; 3 Exercise Physiology, Kent State University, Kent, OH, USA; 4 Department of Exercise Science, Walsh University, North Canton, OH, USA

**Keywords:** bicycling, exercise-induced muscle damage

## Abstract

**Introduction:**

Our objective was to design an eccentric bicycle design to elicit delayed onset muscle soreness (DOMS).

**Methods:**

To assess the bicycle designs’ ability to elicit DOMS, fourteen, recreationally active, males performed five-minutes of eccentric bicycling at 50% of their individualized power determined from a modified six-second Wingate test. Outcome measures to assess DOMS included the Likert pain scale, creatine kinase, lactate blood concentration, and pressure algometry detection evaluated at four time points (baseline (before the eccentric bicycling), immediate post, 24 hours post, and 48 hours post).

**Results:**

The Likert pain scale was different (F = 75.88, p < 0.001) at baseline (0.14 ± 0.36) and immediate post (0.21 ± 0.43), compared to 24 hours post (3.07 ± 0.83), and 48 hours post (2.93 ± 1.07). No changes were reported for creatine kinase (F = 0.7167, p = 0.475), lactate blood concentration (F = 2.313, p = 0.107), or pressure algometry detection.

**Conclusions:**

To understand mechanisms of DOMS, there is a need for a consistent, reliable method for producing DOMS. Our eccentric bicycle design and protocol offers an alternative approach to previous eccentric ergometer designs - demonstrating the potential to elicit DOMS in one, five-minute session.

## Introduction

The reported benefits of eccentric exercise combined with limited commercial options have inspired custom laboratory eccentric ergometer designs [1]. However, compared in other submaximal eccentric bicycling investigations [1–3], our design was approach with the intent to elicit delayed onset muscle soreness (DOMS) in a controlled laboratory setting to better understand its effects.

DOMS is a sensation of discomfort in the skeletal muscle following unaccustomed physical activity involves eccentric muscle contractions [4]. Moreover, DOMS can adversely affect muscular performance due to voluntary reduction of effort and inherent loss of muscle capacity to produce force [5] that temporally produces metabolites and by-products of tissue damage in the body. These metabolites result in inefficient blood supply, and lack of oxygen in the contracting muscle, leading to pain, muscle weakness, decline of range of motion, and reduction of proprioceptive ability [6].

Thus, the purpose of our work is to describe and assess an eccentric bicycle design as a potential method of eliciting DOMS. The central hypothesis was that the eccentric bicycle design would elicit DOMS following a one, five-minute eccentric exercise protocol, supported by increases in creatine kinase, lactate production, and the Likert scale 24–48 hours after eccentric exercise.

## Scientific Methods

A cross-sectional study design was used to assess an eccentric bicycle design to elicit DOMS. Recreationally active, health males ages 18–40 years old were recruited through approved flyers and advertisements. All individuals who met the inclusion criteria completed the American Heart Association/American College of Sports Medicine exercise pre-participation questionnaire [7], and were free of contraindications to exercise including cardiovascular disease (heart attack, heart surgery, angioplasty, pacemaker, rhythm disturbance, heart valve disease, heart failure, heart transplantation, and congenital heart disease), and stroke. Individuals that classified as high risk were excluded from study participation. The study design was approved by the Kent State University Institutional Review Board and research was conducted in accordance with the principles of the Belmont Report.

Participants came to the Exercise Physiology laboratory at Kent State University for four visits (Table 1). On visit one, baseline measures were evaluated. Before visit two, participants were instructed refrain from high-intensity exercise for two weekend days. On visit two, an eccentric bicycle protocol was performed with the intent to elicit DOMS. Follow-up measures were conducted 24 and 48 hours after the eccentric bicycle protocol.

The eccentric bicycle protocol intensity was determined via a modified six second Wingate test on a Velotron ergometer (Quarq Technology, Spearfish, SD, USA). 50% of the individualized peak power (e.g. maximum watts) obtained was used as the target goal for the eccentric bicycle protocol at visit two. During the modified Wingate test, participants wore clipped in cycling shoes (Trek Bontrager, Waterloo, WI, USA) with Shimano pedaling dynamics cleats (Sakai, Japan) to determine peak anaerobic power and anaerobic capacity by pedaling at maximum speed against a constant force.

To determine fitness, participants performed a bicycle maximal oxygen consumption (VO_2_ max) test. The test intensity stated at 30 watts and, increased in increments of 30 watts every minute until participants reached the test criteria while maintaining a self-selected ± 5 revolutions per minute (RPM) on the Velotron ergometer. Expired air was collected and analyzed with a Parvomedics metabolic cart (Parvomedics, Provo, UT, USA) to determine VO_2_. In addition, study staff collected additional supportive outcomes including heart rate through a Polar Wearlink® monitor (Polar Electro Inc., Bethpage, NY, USA), respiratory exchange ratio and, ventilatory threshold through the Parvomedics metabolic cart, and watts cycled recorded through the Velotron computer software [8].

### Eccentric Bicycle Design.

The bicycle was a modified stationary ergometer (ProForm® recumbent cycle frame, Logan, UT, USA) powered by a servo motor fitted to pedal the bike backwards, while participants pedaled forward in an attempt to overcome the force of the motor. The recumbent design had a stable base and a seat position ideal for torso stabilization for eccentric bicycling [1]. Moreover, the bicycle components were replaced and outfitted with Shimano bicycle cranks (175 mm FC-M552, Sakai, Japan), commercially available industrial chain, bearings, and 58 by 22 tooth cogs. The industrial cog required a bolt pattern machined for installation to the eccentric bicycle performed by the Kent State University machine shop (see Image 1C). The eccentric bicycle was outfitted with a servo motor (Servofit Precision Planetary Gearheads, Maysville, KY, USA), and a programmable logic controller (PLC) called Control Logix (Rockwell Automation, Twinsburg, OH, USA). In front of participants was an operator display and control input touch screen, the PanelView Plus graphic display (Rockwell Automation, Twinsburg, OH, USA) as seen here (see Image 1B).

### Eccentric Bicycle Protocol.

Prior to beginning the eccentric bicycle protocol, participants had a familiarization ride and warm-up on the eccentric bicycle for approximately 2–3 minutes performed at 40 RPM. After the warm-up, participants stopped and were provided instructions for the eccentric bicycle protocol which began with a pedaling increase from 0 to 40 RPM within 15–20 second period. Once participants reached 40 RPM, they were instructed to maintain 40 RPM for five minutes with visual feedback provided through the PanelView Plus display. Eccentric bicycle outcomes included RPM collected through RSview Enterprise File Viewer in second interval samples (Rockwell Automation, Twinsburg, OH, USA). To assess the effects of the eccentric bicycle protocol, Likert pain scale, creatine kinase, blood lactate concentration, muscular pain was recorded at all four visits (baseline, eccentric bicycle protocol, 24 hours post, and 48 hours post).

### Anthropometric Measures.

During the first visit, participants’ height (DigiStad HM210D stadiometer, Charder Medical, Tiachung City, Tiawan) weight (Physician Balance Beam scale, Health o meter® Professional, McCook, IL, USA) were measured and body mass index was calculated.

### Likert Pain Scale.

The Likert scale was used to evaluate participants’ level of muscle soreness over the past 12 hours on a seven point scale in which zero represented a complete absence of soreness and six represented a severe pain that limits their ability to move.

### Creatine Kinase.

A blood sample (5 mL) was collected through standard venipuncture technique from the antecubital vein to analyze plasma creatine kinase. Samples were obtained by either trained technicians from the Exercise Physiology program, or the Robinson Memorial Hospital hematology laboratory (Ravenna, OH, USA). All samples were centrifuged at 1,500 revolutions per minute (RPM) for 10 minutes following standard guidelines. Samples were transported to the hematology laboratory for processing.

### Lactate Blood Concentration.

Blood lactate concentration was measured using a blood lactate strip and finger prick assessment (Lactate Plus Nova Biomedical, Waltham, MA, USA).

### Muscle Pain.

Muscular pain was assessed through the self-reported visual analog scale (VAS) and pressure algometry (Wagner FDX, Greenwich, Connecticut, USA). Participants were instructed to report their perceived pain detection and pain threshold and record the algometry on a VAS that consisted of a 15 cm line in which zero represented no pain and 15 represented pain as bad as it could possibly be. Since the eccentric bicycle is a multi-joint design, measures for the hamstring and rectus femoris were also evaluated. Pressure algometry was applied with increasing the pressure slowly and continuously (1 kg/sec) at the center of the muscle belly for the gastrocnemius, hamstring and rectus femoris muscles [9], reporting both pressure detection and pain threshold [10].

Although DOMS may result in discomfort, participants were informed that they may experience muscle soreness or pain lasting approximately 72 hours after the eccentric bicycle protocol [11]. In addition, during the eccentric bicycle protocol, an emergency stop button installed on the PLC and was available for participants and/or study staff to remove power from the servomotor. Moreover, adherence or attendance to all timepoints was closely monitored throughout the investigation.

G*Power analysis software estimated that a sample size of 14 would elicit 0.8 power and 0.99 effect size [12]. All data was analyzed using GraphPad Prism 8.4.1 (GraphPad Software, San Diego, CA, USA). Alpha was set *apriori* p ≤ 0.05. Descriptive statistics (mean ± standard deviation) are reported for participant baseline characteristics, self-reported muscle pain detection and threshold. Repeated measures one-way analysis of variance evaluated Likert pain, creatine kinase, blood lactate concentration, and pressure algometry detection across timepoints with Tukey’s multiple comparisons test.

## Results

Participants (N = 14) were young, healthy males (21.14 ± 2.25 years old; 23.64 ± 2.22 kg/m^2^ body mass index), recreationally active runners (7.8 ± 5.6 miles week). Fitness levels were assessed and participants had mean baseline VO_2_ max of 40.56 ± 8.07 ml/kg/min, and peak Wingate power of 737.93 ± 165.90 watts. Approximately 10% of participants completed the baseline (T1) visit, but were categorized as lost to follow-up and did not complete T2-T4 visits. Their baseline (T1) data was not included in outcome measures.

**Figure 1.**
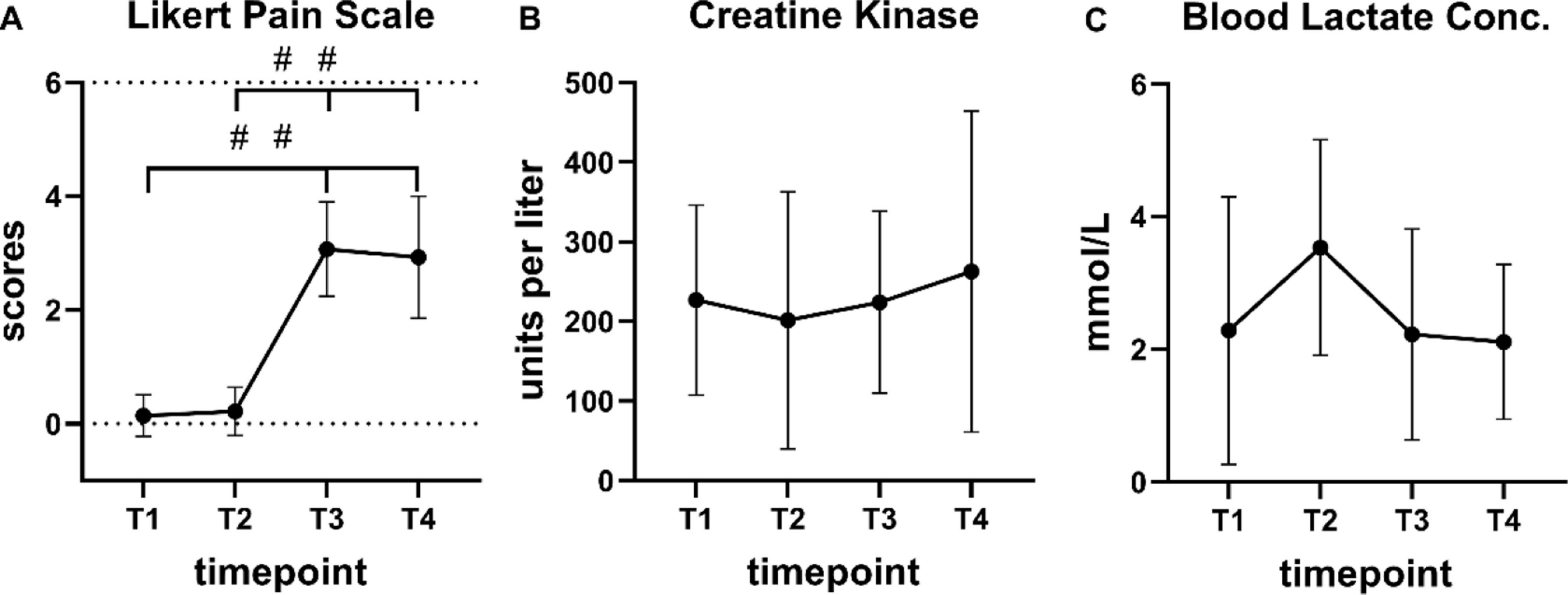
Values represent the Likert pain scale (A), creatine kinase (B), and blood lactate concentration (C), before and after the eccentric bicycle protocol. Data are presented as mean ± SD. # < 0.001 p value. T1 = baseline, T2 = eccentric bicycle protocol, T3 = 24 hours post, T4 = 48 hours post.

The Likert pain scale (Figure 1A) was significantly different over time (F = 75.88, *p* < 0.001) with the following mean changes (T1: 0.14 ± 0.36; T2: 0.21 ± 0.43; T3: 3.07 ± 0.83; and T4: 2.93 ± 1.07). T1 and T2 were both significantly different from T3 and T4, respectively (*p* < 0.001). Both creatine kinase (Figure 1B) was not statistically significant over time (F = 0.717, *p* = 0.475), as well as lactate (Figure 1C) was not statistically different over time (F = 2.313, *p* = 0.107).

**Figure 2.**
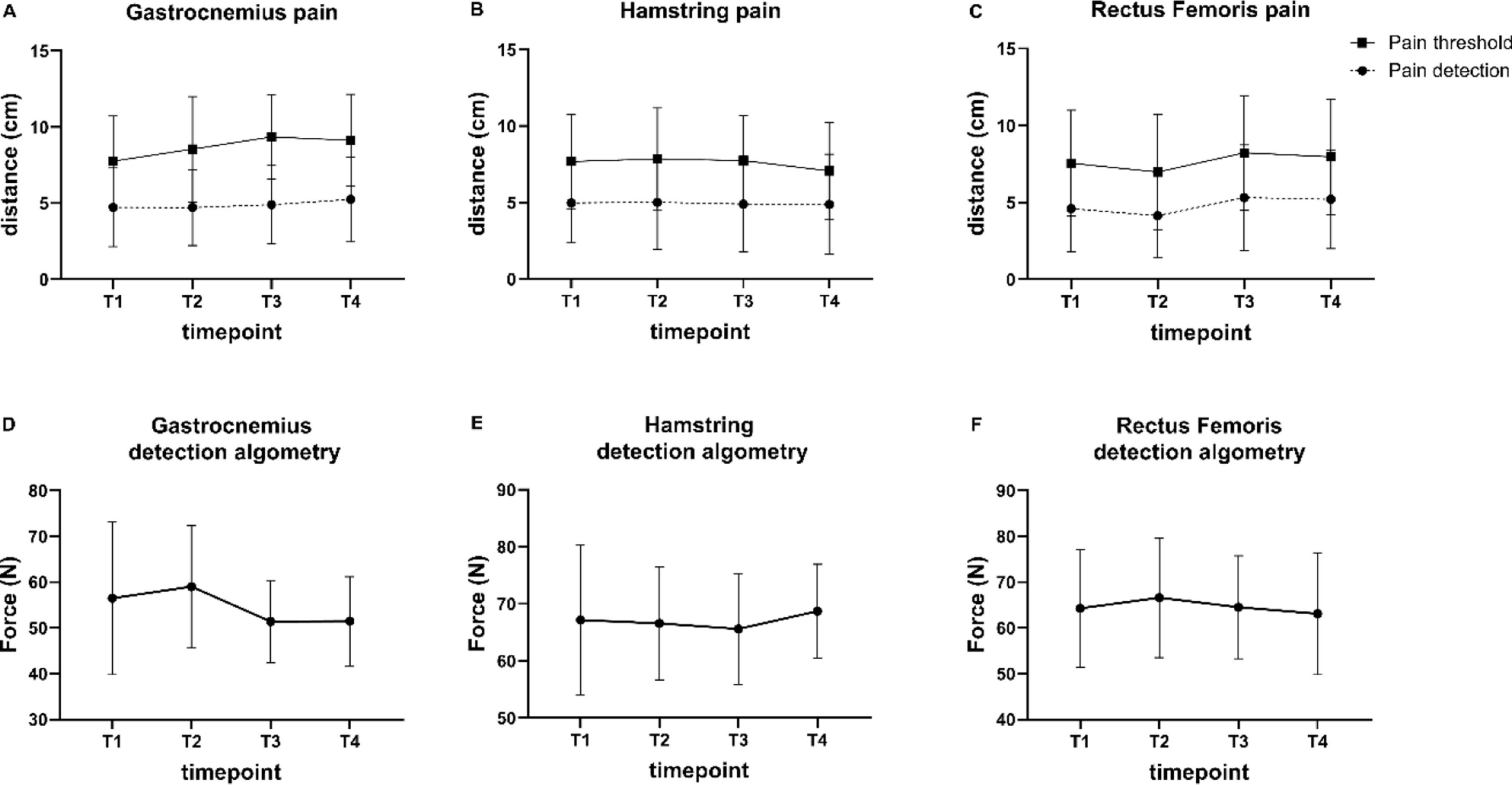
Values represent muscular self-reported visual analog scale distance for pain detection and threshold for the gastrocnemius (A), hamstring (B), and rectus femoris (C) before and after the eccentric bicycle protocol. The scale consisted of a 15 cm line with zero representing no pain and 15 representing pain as bad as it could possibly be. Algometry detection results are presented for the gastrocnemius (D), hamstring (E), and rectus femoris (F) muscles. Data are presented as mean ± SD. There were no significant differences across timepoints. T1 = baseline, T2 = eccentric bicycle protocol, T3 = 24 hours post, T4 = 48 hours post.

Pain detection and threshold descriptive statistics for the gastrocnemius, hamstring, and rectus femoris muscles (Figure 2A-C) and pressure detection algometry changes over time (Figure 2D-F) are presented. Gastrocnemius pressure algometry detection was not significantly different across all timepoints (F = 2.054, *p* = 0.1608). Similar results were found for hamstring pressure algometry (F = 0.4637, *p* = 0.6558), and rectus femoris algometry (F = 0.5985, *p* = 0.5748).

## Discussion

The purpose of our technical note is to describe and assess an eccentric bicycle design as a reliable method of eliciting DOMS. The central hypothesis was that DOMS would occur following our eccentric bicycling protocol, supported by increases in creatine kinase, lactate production, and pain 24–48 hours after eccentric bicycling. Our approach was not without its limitations – the eccentric bicycle design did not utilize a power meter which has been incorporated in other versions of eccentric bicycle designs. While this may be a limitation, a power meter was not absolutely necessary to investigate the onset of DOMS. Moreover, our protocol varied from earlier investigations that were designed to assess submaximal (low to moderate intensity) eccentric bicycling, to avoid the onset of DOMS [1–3]. Specifically, Elmer et al. methodology instructed participants to maintain 60 RPM at 20% of maximum concentric power for five minutes [13], while our primary goal was to elicit DOMS in one, five-minute bout.

In our investigation participants reported significant differences in the Likert pain scale from T1 and T2 with increases in pain reported 24 (T3) and 48 (T4) hours post eccentric bicycling. These findings are aligned with previous onset of DOMS results with soreness developing 48 hours post exercise [14–16]. Our results also indicated that creatine kinase and lactate blood markers exhibited no changes over time. Previously, creatine kinase has been described as having larger inter-individual response variation [17], and thus not strongly correlated with muscle damage noting that morphological changes may be greater among older adults [18]. Lactate blood concentration did not exhibit changes across the timepoints. However, the results immediate post eccentric bicycling (T2) was likely influenced by a potential delay in blood collection that ranges from immediate post to 20 minutes post eccentric bicycling. In previous investigations, blood lactate concentration was determined immediately post eccentric exercise [19]. Moreover, no differences were reported in pressure algometry. Although historically, algometry has been considered a relatively reliable method to assess experimental induced DOMS, Nussbaum (1998) noted that pressure algometry measures may not be precise [10].

Our eccentric bicycle design and protocol offers an alternative approach to previous eccentric ergometers and protocols [1–3] that were designed with the intent to avoid DOMS, training at low to moderate intensities. Moreover, it demonstrates the potential to elicit DOMS in one, five minute session individualizing the desired power output based on 50% of participants’ modified Wingate performance. To understand mechanisms of DOMS, there is a need for a consistent, reliable method for producing DOMS and an eccentric bicycling design may be a potential approach.

## Conclusions

To understand mechanisms of DOMS, there is a need for a consistent, reliable method for producing DOMS. Our eccentric bicycle design and protocol offers an alternative approach to previous eccentric ergometer designs - demonstrating the potential to elicit DOMS in one, five-minute session.

## Figures and Tables

**Images.**
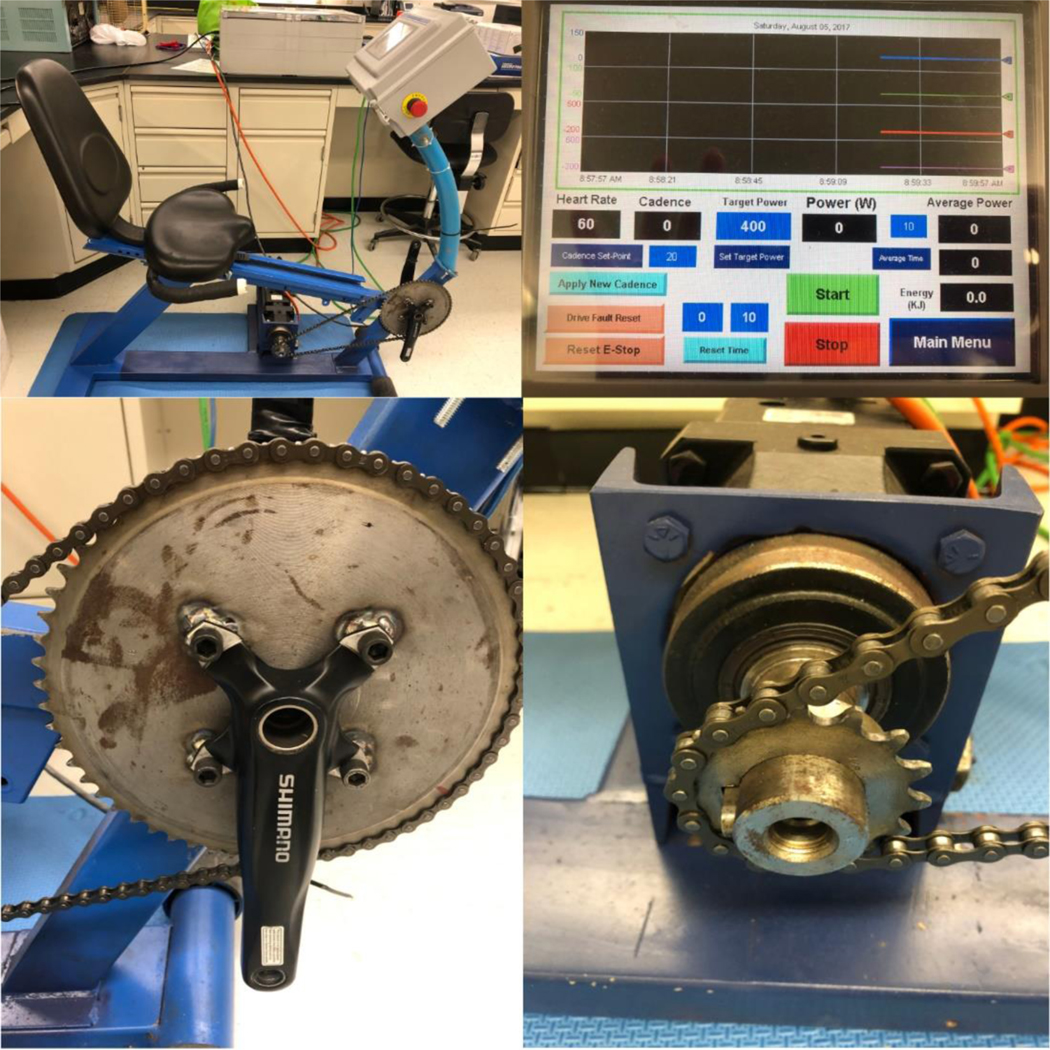
Note. Side view of the custom eccentric bicycle (A, upper left). Participants point of view of the operator display (B, upper right). Industrial cog used (C, lower left). 22 tooth cog attached to the servomotor (D, lower right).

**Table 1.** Study Protocol

Study Phase	T1	T2	T3	T4
Informed Consent, Inclusion/Exclusion	X			
Anthropometric Measures	X			
Maximal Oxygen Consumption Test	X			
Modified Wingate Test	X			
Eccentric Bicycling		X		
Creatine Kinase	X	X	X	X
Lactate Test	X	X	X	X
Likert Scale	X	X	X	X
Muscle Pain (algometry and self-reported)	X	X	X	X
Muscle Length	X	X	X	X

Note. T1 = baseline, T2 = eccentric bicycle protocol, T3 = 24 hours post, T4 = 48 hours post.
